# Editorial: Human-Animal Interaction (HAI) Research: A Decade of Progress

**DOI:** 10.3389/fvets.2020.00044

**Published:** 2020-02-18

**Authors:** Sandra McCune, Peggy McCardle, James A. Griffin, Layla Esposito, Karyl Hurley, Regina Bures, Katherine A. Kruger

**Affiliations:** ^1^Waltham Centre for Pet Nutrition, Melton Mowbray, United Kingdom; ^2^PM Consulting LLC, Tarpon Springs, FL, United States; ^3^Haskins Laboratories, New Haven, CT, United States; ^4^Eunice Kennedy Shriver National Institute of Child Health and Human Development (NICHD), Bethesda, MD, United States; ^5^Mars Inc, McLean, VA, United States; ^6^School of Veterinary Medicine, University of Pennsylvania, Philadelphia, PA, United States

**Keywords:** human-animal bond, human-animal interaction (HAI), human-animal relationships, child development, animal assisted intervention, animal assisted activities

In 2008, the *Eunice Kennedy Shriver* National Institute of Child Health and Human Development (NICHD), one of the 27 Institutes and Centers that make up the U.S. National Institutes of Health (NIH), and the WALTHAM® Centre for Pet Nutrition (WALTHAM®), a division of Mars Inc., entered a public-private partnership (henceforth referred to as “the Partnership”) to explore the science of human-animal interaction (HAI), specifically as it relates to children's health and overall development. To take stock of existing research and more fully understand research needs in this area, the partners convened a series of workshops, bringing together researchers and practitioners currently working in HAI as well as individuals with expertise in other relevant fields including ethology, developmental, cognitive, clinical and comparative (animal) psychology, pediatric and veterinary medicine, epidemiology, and public health. Based on the research needs identified, the NICHD established a new research program on HAI and child health and development (1).

Those initial meetings also led to the publication of two edited volumes to disseminate the information from the presentations and the rich discussions that took place. The first volume, *Animals in Our Lives* (2), provided information about HAI research design and methodology and studies of HAI in child development and human health. The second, *How Animals Affect US* (3), had a more applied focus, highlighting HAI in family, community, and therapeutic settings and emphasizing the need for evidence-based practice in this relatively young and rapidly growing research area.

Informed by the workshops, NICHD, joined by the NIH's National Institute of Nursing Research, issued the first in a series of Funding Opportunity Announcements (FOAs) (1) partially funded through a gift to the NICHD from Mars, Incorporated under the Partnership. The first FOAs focused on how children perceive, relate to, and think about animals; how pets in the home impact children's social and emotional development and health (e.g., allergies, asthma, mitigation of obesity); and whether and under what conditions therapeutic use of animals is safe and effective. Seven research grants were funded under the initial FOAs, and between 2008 and 2018 the NICHD has issued 8 FOAs and funded a total of 27 research grants in HAI (for current information on funded grants and current FOAs see https://www.nichd.nih.gov/about/org/der/branches/cdbb/programs/psad/HAI).

As the research investments began to bear fruit the Partnership continued to evolve and new Research Topics emerged involving a wider range of scientific fields. Researchers were documenting the relationship between the impact of HAI and child development, but little was known about the mechanisms underlying these effects. In 2011, a workshop was held on the social neuroscience of HAI, which formed the basis for another edited volume, *The Social Neuroscience of Human-Animal Interaction* (4), addressing the basic neurobiological mechanisms that underlie the effects observed in HAI. The Partnership also continued to disseminate information regarding new directions in HAI research (5) as well as how new research findings were relevant to clinical applications such as Animal-Assisted Therapy (6, 7) and HAI in school settings (8).

These Partnership workshops, research solicitations, and publications over the past 10 years have promoted the adoption and use of more rigorous designs and methods, raising the bar for the quality of research in HAI. As a result, the field has broadened the scope of studies undertaken, addressing not only correlational studies examining the association between interactions with pets and the subsequent social-emotional benefits generally, but also potential mechanisms for those quantifiable effects, including intervention studies employing randomized controlled trial designs.

In addition to promoting randomized controlled trials, the NICHD-Mars Partnership also has recognized the need to encourage and support more public health research through population representative studies. These efforts have included the development of survey questions/instruments and their inclusion in ongoing large-scale cross-sectional surveys and longitudinal studies that use population representative samples (5).

Embedding pet ownership questions in these studies informs our knowledge of the prevalence of pets and offers a cost-effective way to collect data on the impact of pets on health and development for children, adults, and families for secondary analyses. Because data from these studies are publicly available, this also increases the research resources for the field of HAI. While cross-sectional data help describe the relationships between pet ownership and health and development, the Partnership's goal is, where possible, to encourage longitudinal data collection on the same individuals to allow for the study of changes in pet ownership over time and concomitant changes in owner physical and mental health status and overall well-being.

The Partnership also has fostered an increased focus on the health benefits of interaction with animals, including therapeutic and rehabilitative interventions for children and adults with intellectual, developmental, physical, and mental health-related disabilities and other disorders. These studies frequently examine the effects of HAI interventions on both the humans and the animals involved, exploring how the animals may benefit but also suffer stress as the result of their role in the intervention. Such studies are needed to identify appropriate selection, training, and monitoring protocols to ensure the well-being of the animal and the optimal conditions for the therapeutic interaction with the person receiving the intervention.

There is ample evidence that the field of HAI has grown and matured over the last decade (9), including the increased number of academic positions in research and education, the development of HAI Centers of expertise (e.g., Tufts' s Institute for HAI and Purdue's Center for the Human-Animal Bond), and funding and industry support through non-governmental organizations such as the Human-Animal Bond Research Institute (HABRI) and the Horses and Humans Research Foundation (HHRF). Likewise, journals dedicated to HAI research have expanded beyond *Anthrozoos* (a peer-reviewed journal launched in 1987) to include the *HAI Bulletin* in 2013, which resulted from the establishment of the Human-Animal Interaction Section of Division 17 (Society of Counseling Psychology) by the American Psychological Association (APA) in 2012, as well as the creation of a special section devoted to HAI research in the journal *Applied Developmental Science* in 2016.

As recognition of the success of this decade-long collaborative effort, the Research Topic “Human-Animal Interaction: A Decade of Progress” highlights the research jointly funded by the Partnership. This thematic series of original research papers addresses a variety of topics within HAI research. Several papers address children's experiences and relationships with pets (Hart et al.; Hurley and Oakes; Kertes et al.; Meints et al.), one on teaching families to read the body language of dogs (Meints et al.), and an Opinion article highlighting potential health benefits of dog walking even when you are not the owner (Chen). There are reports on the social-emotional effects of pet ownership (Jacobson and Chang), and intervention studies of behavioral and biological effects of animal-assisted interventions on stress (Pendry et al.), including stress-reduction and adaptive behaviors in children with Autism Spectrum Disorder (ASD) (Gabriels et al.; Pan et al.), Attention Deficit Hyperactivity Disorder (ADHD) (Schuck et al.), and incarcerated youth (Syzmanski et al.). Three papers address new measures and methods (Guérin et al.; MacLean and Hare; Bures et al.). The final paper, by members of the NICHD-Mars Partnership (Griffin et al.) addresses what will be necessary to sustain and accelerate progress in HAI research over the coming decade. We note that this Research Topic does not reflect all of the research of all the investigators funded under the Partnership, as many have already published their findings and others are still in the data collection phase of their projects.

## Author Contributions

All co-authors contributed to the design and writing of the paper. SM, PM, and JG served as Editors for the Research Topic. LE, KH, RB, and KK were not Topic Editors of this Research Topic but co-wrote the Editorial.

### Conflict of Interest

SM and KH are employed by Mars Incorporated. KK was supported by a grant from Mars Inc. PM was employed by PM Consulting and as a science and ethics consultant by Mars Inc. The remaining authors declare that the research was conducted in the absence of any commercial or financial relationships that could be construed as a potential conflict of interest.
